# Evaluating the Impact of Enteral Nutrition vs Parenteral Nutrition on Postoperative Outcomes in Esophageal Cancer Patients After Esophagectomy: A Systematic Review and Meta-Analysis of Randomized Controlled Trials

**DOI:** 10.7759/cureus.92413

**Published:** 2025-09-15

**Authors:** Ahmed Gamal Abouarab, Ahmed Hamdy Mohsen, Noha E Mosellhy, Mohamed Ibrahim Mousa, Omar Abbas, Hashim Manea

**Affiliations:** 1 Intensive Care Unit, Ain Shams University Hospitals, Cairo, EGY; 2 Surgery, Oregon Health & Science University, Portland, USA; 3 College of Medicine, University of Warith Al-Anbiyaa, Karbala, IRQ

**Keywords:** enteral nutrition, esophageal cancer, meta-analysis, parenteral nutrition, systematic review

## Abstract

Esophageal cancer is a major global health issue, with high incidence, morbidity, and mortality rates. Esophagectomy is a common treatment option; however, it may lead to multiple postoperative complications, which carry significant risks for patients’ nutritional and immunological status. While adequate nutritional support has been postulated to play a significant role in reducing postoperative morbidity and mortality in esophageal cancer patients, no consensus has been reached regarding the optimal approach. We have conducted this meta-analysis in an attempt to bridge this gap by assessing the effects of enteral nutrition (EN) versus parenteral nutrition (PN) on postoperative recovery and outcomes in these esophageal cancer patients following esophagectomy. A systematic search of PubMed, Scopus, Web of Science, Scholar, and Cochrane Central Register of Controlled Trials was conducted from inception till March 12, 2025. Our inclusion criteria were: (i) randomized controlled trials; (ii) esophageal cancer patients post-esophagectomy; (iii) EN only compared to PN only; (iv) nutrition started on the first postoperative day with no preoperative EN or PN; and (v) outcomes should include at least one of the following: postoperative nutritional status, immunological status, total postoperative complications, pulmonary complications, anastomotic leakage, postoperative hospital stay, and mortality. The meta-analysis included six studies with a total of 276 patients. Patients who received EN experienced a greater decrease in albumin levels at POD 7 from baseline (MD 0.12, 95% CI [0.00, 0.25], P = 0.05) compared to patients who received PN. EN was linked to a more pronounced decrease in bilirubin levels at POD 7 from baseline compared to PN (MD -0.69, 95% CI [-1.12, -0.26], P = 0.002). Patients who received PN also had a higher incidence of pulmonary complications (RR 0.46, 95% CI [0.21, 1.00], P = 0.05), total postoperative complications (RR 0.73, 95% CI [0.54, 0.98], P = 0.04) and longer hospital stays (MD -3.91, 95% CI [-4.72, -3.09], P<0.00001). No significant differences were found in prealbumin level changes (MD 0, 95% CI [-0.27, 0.27], CRP levels (MD -1.35, 95% CI [-3.34, 0.64], P = 0.18), total protein levels (MD 0.10, 95% CI [-0.04, 0.24], P = 0.15) anastomotic leakage incidence (RR 1.17, 95% CI [0.39, 3.46], P = 0.78) or mortality (RR 1.59, 95% CI [0.22, 11.60], P = 0.64). EN and PN appear to have distinct effects on esophageal cancer patients after esophagectomy. While EN could result in a significant decrease in albumin levels postoperatively when compared to PN, it is associated with better control of bilirubin levels, a lower incidence of pulmonary and total postoperative complications, and shorter hospital stays. The potential benefits of EN may outweigh its risks. The use of EN compared to PN must be evaluated on an individual basis for each patient. Finally, further research is recommended to explore the reasons behind some outcomes such as the lack of significant differences in pre-albumin levels, total protein levels, CRP levels, anastomotic leakage, and mortality.

## Introduction and background

Esophageal cancer is the eighth most commonly diagnosed cancer and is the sixth leading cause of cancer death worldwide [1,2]. Its incidence and prevalence are higher in developing countries and male gender when compared to developed countries and female gender, respectively [2]. Risk factors that increase the likelihood of having esophageal cancer include alcohol consumption, cigarette smoking, and Barrett's esophagus. Less common risk factors include spicy food, motility disorders such as achalasia (30 times more likely to suffer from esophageal cancer), lye strictures, celiac disease, and Plummer-Vinson syndrome (characteristic triad of dysphagia, Iron deficiency anemia, and esophageal webbing) [3-6]. Symptoms of esophageal cancer, which vary from regurgitation, chest pain not related to eating, progressive dysphagia to solids more than liquids, heartburn, vomiting blood, and weight loss, often emerge late, which can delay diagnosis and worsen prognosis [7]. 

Esophagectomy has been used as one of the mainstay options of treating esophageal cancer [8], but, like any major surgery, it is associated with multiple complications. Typically, early complications include anastomotic leak, atrial dysrhythmias, pneumonia/aspiration, chylothorax, and recurrent nerve palsy. Late complications include dysphagia/stricture, delayed gastric emptying, bile reflux, dumping syndrome, and malabsorption [9]. These complications can have a deteriorating effect on the nutritional and immunological status of the patients postoperatively. Surgical trauma can also lead to intestinal ischemia, atrophy, and increased mucosal permeability, which in turn could lead to bacteremia and sepsis that could not be tackled by the already compromised humoral and cellular immune responses [10]. 

Nutritional support plays a critical role in improving outcomes after esophagectomy. The two primary modalities for postoperative nutrition are EN (enteral nutrition) and PN (parenteral nutrition). Although EN is often preferred for preserving gut integrity and reducing bacterial translocation, PN avoids aspiration risk and may be necessary when the gastrointestinal tract cannot be used. Outcomes that vary across studies include nutritional and inflammatory markers, infection rates, anastomotic leak, length of hospitalization, and mortality. These inconsistencies create ongoing uncertainty about the optimal nutritional strategy after esophagectomy [11,12].

Given the lack of consensus, this meta-analysis aims to discuss the possible benefits of both methods with a detailed comparison of the outcome postoperatively in terms of their impact on the nutritional and immunological status, along with their effect on postoperative morbidity and mortality.

## Review

Methods

This systematic review was conducted in accordance with the guidelines outlined in the “Cochrane Handbook for Systematic Reviews of Interventions”, ensuring methodological rigor and transparency throughout the review process [13].

Search Strategy

We searched PubMed, Scopus, Web of Science, Scholar, and Cochrane Central Register of Controlled Trials from inception till March 12, 2025, using the following terms: Enteral Nutrition [MeSH Terms] AND Parenteral Nutrition [Mesh Terms] AND Esophageal Cancer [MeSH Terms]. We also reviewed the bibliographies of relevant studies to identify any other studies. We only included randomized controlled trials and English-language literature. The initial search and screening were done by two independent authors. Any discrepancies were resolved by discussion between the authors.

Inclusion Criteria

The inclusion criteria were: (i) randomized controlled trials; (ii) esophageal cancer patients post-esophagectomy; (iii) EN only compared to PN only; (iv) nutrition started on the first postoperative day (POD) with no preoperative EN or PN; and (v) outcomes should include at least one of the following: postoperative nutritional status, immunological status, total postoperative complications, pulmonary complications, anastomotic leakage, postoperative hospital stay, and mortality. Pulmonary complications refer to pneumonia, acute respiratory distress syndrome, and atelectasis. Total postoperative complications refer to all complications reported by the studies, including infectious and noninfectious complications. Reported changes in variables refer to changes between POD 7 and baseline (preoperative values). Studies that did not provide the aforementioned variables were excluded. Two independent authors assessed the studies for eligibility based on full text.

Data Extraction

After the studies were selected, two independent authors extracted the data on the first author, publication year, number of patients, approaches of EN and PN, postoperative nutritional support duration, and outcomes using a standardized form. Discrepancies were resolved by discussion between the authors.

Risk of Bias Assessment

Two authors independently assessed the risk of bias using the Cochrane Risk of Bias 2 (RoB 2) tool according to five domains: randomization process, deviations from the intended interventions, missing outcome data, measurement of the outcomes, and selection of the reported result. Each domain was graded as high, some concern, or low risk of bias, and an overall grade was determined using the tool algorithm. Disagreements were resolved by discussion with a third author.

Statistical Analysis

We used Review Manager (RevMan) Version 8.5.1. The Cochrane Collaboration, 2024, available at revman.cochrane.org to perform the statistical analysis. Risk ratios (RRs) were calculated for dichotomous outcomes, and mean differences (MDs) were calculated for continuous outcomes with 95% confidence intervals (CIs) using either a random-effects or a fixed-effects model. A RR was regarded as statistically significant when its 95% CI did not include the value 1, and an MD was considered statistically significant when its 95% CI did not include the value 0. The heterogeneity across pooled studies was assessed using the I2 test. A value of I2 = 25%-50% was considered low, a value of 50%-75% considered moderate, and a value greater than 75% considered high heterogeneity [14]. If significant heterogeneity was present, a random-effects model meta-analysis was used; otherwise, a fixed-effects model was considered, given the small number of studies. To investigate heterogeneity, we also performed sensitivity analysis for the outcomes that had high heterogeneity to identify the source of heterogeneity.

We used a correlation coefficient of 0.99 for imputing the standard deviations of changes at POD 7 from baseline in cases where the standard deviation of changes was not reported, based on the close matches with the small SDs reported in similar studies reporting the same variables. This high correlation coefficient was chosen because the study conditions were highly controlled and the follow-up period was short. After using our correlation coefficient, multiple correlation coefficients were used to examine the sensitivity and robustness of the analysis, as stated in the 16.1.3.2 section of the Cochrane Handbook for Systematic Reviews of Interventions [13].

Results

Study Characteristics

**Figure 1:**
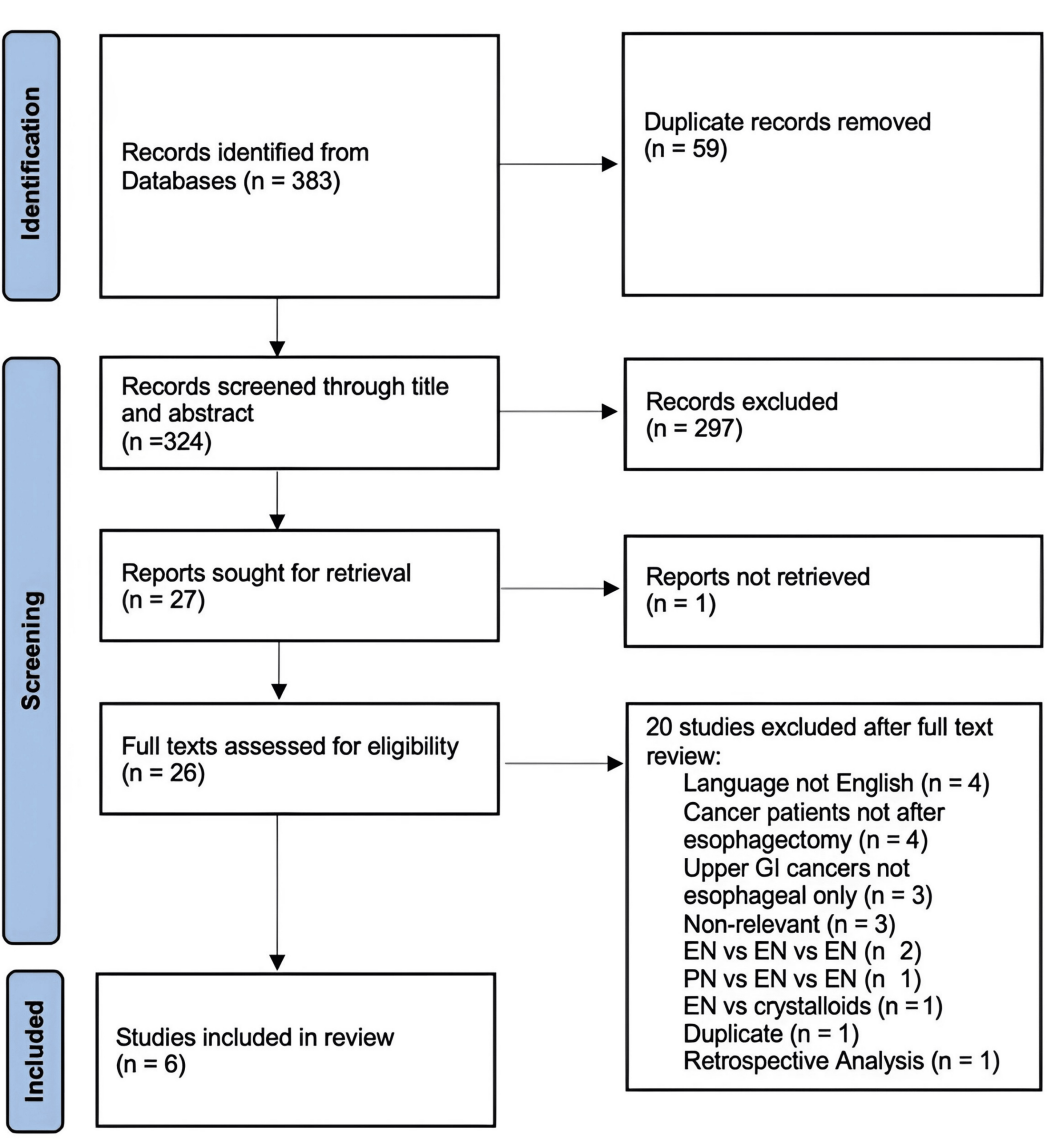
PRISMA flowchart PRISMA: Preferred Reporting Items for Systematic Reviews and Meta-Analyses

Our initial search resulted in 383 records. After duplicates were removed, 324 records were screened through title and abstract. Initial screening resulted in 27 studies being sought for retrieval. One study was not retrieved as the full text was not available, then we assessed the full texts of the other 26 studies for eligibility. We excluded 20 studies for the reasons mentioned in the Preferred Reporting Items for Systematic Reviews and Meta-Analyses (PRISMA) flowchart (Figure 1). Finally, six studies were included in this meta-analysis [15-20].

Study characteristics of the six included studies are summarized in Table 1. All of the studies are RCTs. A total of 276 patients were included in our meta-analysis with varying sample sizes. The sample sizes, EN and PN approaches, nutritional support (NS) durations and outcomes for each study are listed in Table 1. Regarding nutritional status, six studies reported changes in albumin levels, four reported changes in prealbumin levels, four reported changes in total protein, and three reported changes in bilirubin levels. Four studies also reported changes in CRP levels. Regarding complications, four studies reported pulmonary complications, four reported anastomotic leakage, and six reported total postoperative complications. Finally, three studies reported postoperative hospital stay durations and three studies reported mortality rates.

**Table 1:** Characteristics of the included studies ‡Nutritional support duration; † Central venous catheter; §Postoperative day

First Author	Year	Sample Size (E/P)	EN Approach	PN Approach	NS Duration^‡^	Outcomes
Hamai [15]	2021	19/21	Jejunostomy	CVC^†^	POD^§^ 1-7	Albumin, Total Protein, Total postoperative complications
Takesue [16]	2015	24/23	Jejunostomy	CVC^†^	POD^§^ 1-14	Albumin, Prealbumin, Total Protein, Bilirubin, CRP, Pulmonary complications, Anastomotic leakage, Total postoperative complications, Postoperative hospital stay, Mortality
Mashhadi [17]	2015	20/20	Jejunostomy	CVC^†^	POD^§^ 1-7	Albumin, Prealbumin, Total Protein, CRP, Pulmonary complications, Anastomotic leakage, Total postoperative complications, Mortality
Yu [18]	2013	50/46	Nasoduodenal feeding tube	CVC^†^	POD^§^ 1-7	Albumin, Total Protein, Bilirubin, Pulmonary complications, Total postoperative complications, Postoperative hospital stay, Mortality
Seike [19]	2011	14/15	Nasojejunal feeding tube	CVC^†^	POD^§^ 1-7	Albumin, Prealbumin, CRP, Total postoperative complications
Aiko [20]	2001	13/11	Jejunostomy	CVC^†^	POD^§^ 1-7	Albumin, Prealbumin, Bilirubin, CRP, Pulmonary complications, Anastomotic leakage, Total postoperative complications, Postoperative hospital stay

Risk of Bias

**Figure 2:**
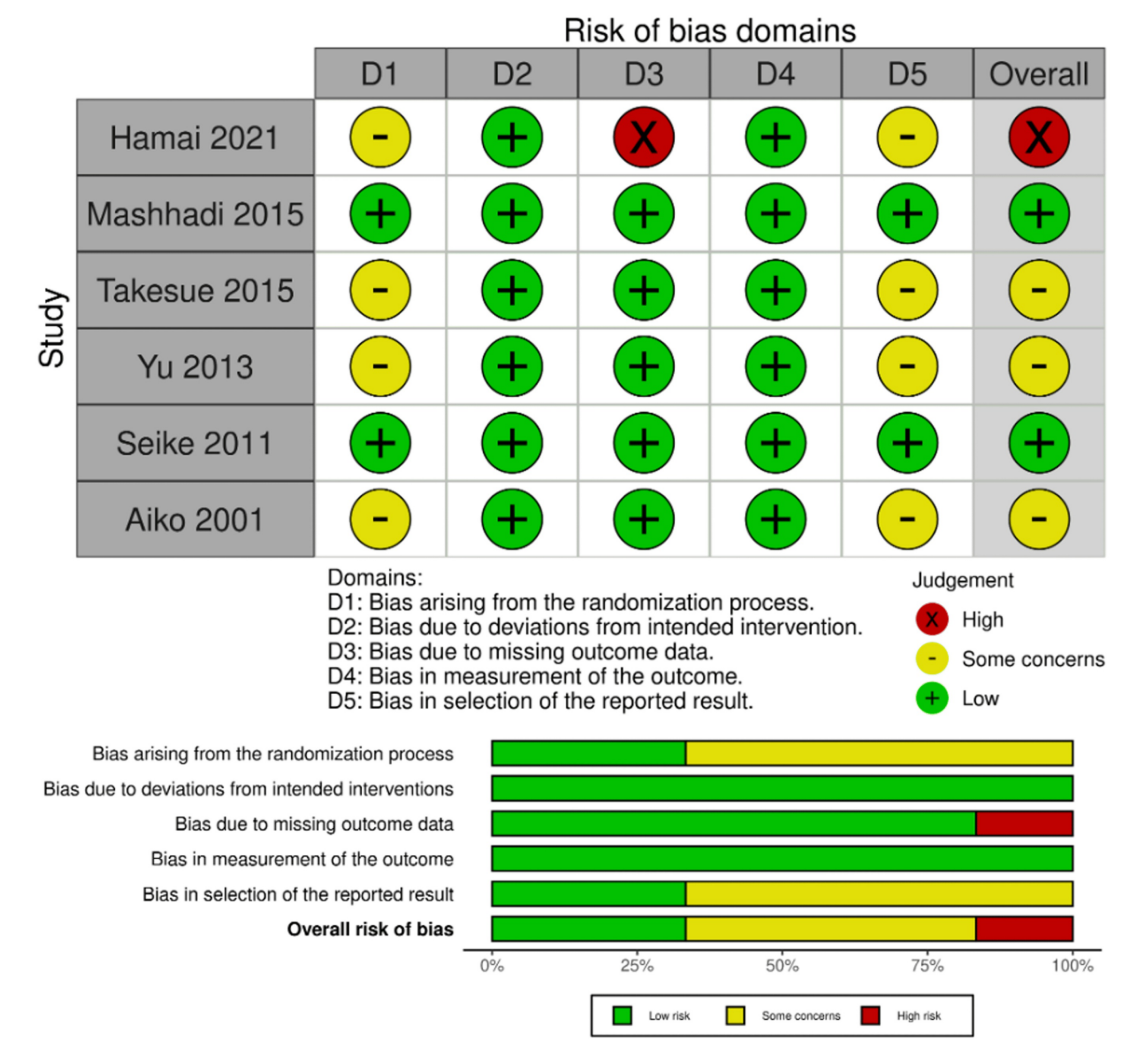
Risk of bias summary Hamai 2021 [15], Takesue 2015 [16], Mashhadi 2015 [17], Yu 2013 [18], Seike 2011 [19], Aiko 2001 [20]

We used the ROB2 tool to assess the risk of bias, and the results are presented in Figure 2. We judged that most of the individual domains in all studies showed low risk of bias. One study, however, showed high risk of bias in one domain, which by algorithm definition, yields an overall high risk of bias for this study. The high risk of bias in the third domain in this study (Hamai 2021, [15]) stems from missing outcome data, as several patients were excluded from both the enteral nutrition and parenteral nutrition groups due to complications. This raises concerns about the reliability of the study's findings and the overall completeness of the data, potentially affecting the conclusions drawn from the analysis. Three studies showed overall some concerns for bias and two studies revealed overall low risk of bias. Figure 2 summarizes the risk of bias in the six studies. We did not perform analysis for publication bias as the funnel plot or any other statistical method does not have enough power to detect bias in such a small number of studies.

Nutritional Markers

**Figure 3:**
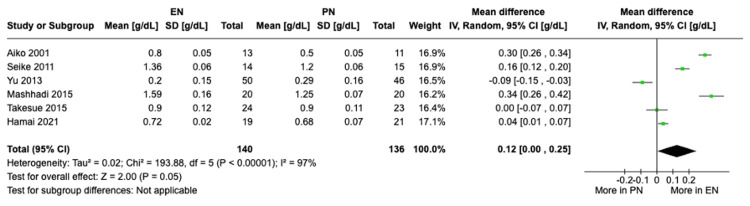
A forest plot comparing a decrease in albumin levels between EN and PN Hamai 2021 [15], Takesue 2015 [16], Mashhadi 2015 [17], Yu 2013 [18], Seike 2011 [19], Aiko 2001 [20] EN: Enteral nutrition; PN: Parenteral nutrition

Decrease in albumin levels: A meta-analysis of six studies with a total of 276 patients showed a significant difference in the decrease in albumin levels from preoperative baseline to POD 7 between the EN and PN groups (Figure 3). Patients receiving EN experienced a statistically significant greater decrease in albumin levels at POD 7 from baseline compared to those receiving PN under the random-effects model (MD 0.12, 95% CI [0.00, 0.25], P = 0.05). The analysis showed high heterogeneity (I² = 97%). Sensitivity analysis showed consistently high heterogeneity after exclusion of each of the individual studies.

**Figure 4:**
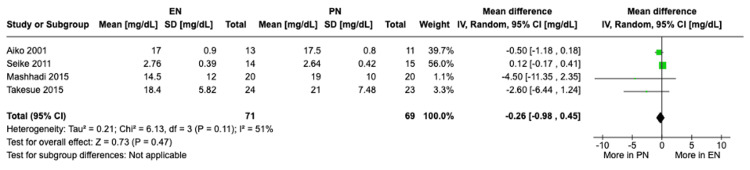
A forest plot comparing the change in prealbumin levels between EN and PN Hamai 2021 [15], Takesue 2015 [16], Mashhadi 2015 [17], Yu 2013 [18], Seike 2011 [19], Aiko 2001 [20] EN: Enteral nutrition; PN: Parenteral nutrition

Change in prealbumin levels: Four studies with a total of 140 patients showed no significant difference in changes in prealbumin levels from preoperative baseline to POD 7 between the EN and PN groups under the random-effects model (MD -0.26, 95% CI [-0.98, 0.45], P = 0.47) (Figure 4). A fixed-effects model also showed no statistically significant difference (MD 0, 95% CI [-0.27, 0.27], P = 0.99). Moderate heterogeneity was observed (I² = 51%). Upon excluding the study by Seike et al. (2011) [19], heterogeneity dropped to I2=16%; however, still no statistically significant results were observed upon secondary analysis.

**Figure 5:**
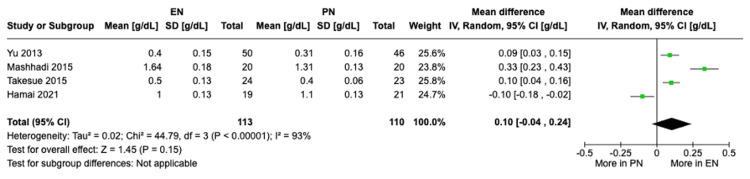
A forest plot comparing the decrease in total protein levels between EN and PN Hamai 2021 [15], Takesue 2015 [16], Mashhadi 2015 [17], Yu 2013 [18], Seike 2011 [19], Aiko 2001 [20] EN: Enteral nutrition; PN: Parenteral nutrition

Decrease in total protein: A meta-analysis of four studies with a total of 123 patients was done using the random-effects model. It showed no statistically significant difference between the two methods of feeding in reduction of total protein levels from preoperative baseline to POD 7 (MD 0.10, 95% CI [-0.04 ,0.24], P = 0.15) (Figure 5). High heterogeneity was observed (I² = 93%). Sensitivity analysis showed consistently high heterogeneity after exclusion of each of the individual studies.

**Figure 6:**
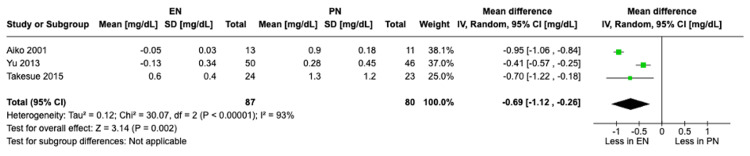
A forest plot comparing the change in bilirubin levels between EN and PN Hamai 2021 [15], Takesue 2015 [16], Mashhadi 2015 [17], Yu 2013 [18], Seike 2011 [19], Aiko 2001 [20] EN: Enteral nutrition; PN: Parenteral nutrition

Change in bilirubin: A meta-analysis of three studies with a total of 167 patients showed a significant difference in the change in bilirubin levels at POD 7 compared to baseline in both groups. The PN group experienced a greater increase in bilirubin levels compared to the EN group at POD 7 from baseline. This increase was statistically significant (MD -0.69, 95% CI [-1.12, -0.26], P = 0.002) (Figure 6).

Inflammatory Markers

**Figure 7:**
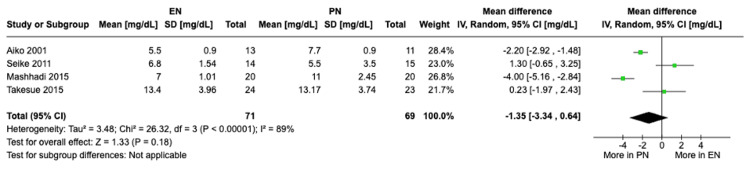
A forest plot comparing the increase in CRP levels between EN and PN Hamai 2021 [15], Takesue 2015 [16], Mashhadi 2015 [17], Yu 2013 [18], Seike 2011 [19], Aiko 2001 [20] EN: Enteral nutrition; PN: Parenteral nutrition

Increase in C-reactive protein levels: A meta-analysis of four studies with a total of 140 patients was done using random-effects models. Patients receiving PN were shown to have a higher increase in CRP levels compared to patients receiving EN, although not statistically significant (MD -1.35, 95% CI [-3.34, 0.64], P = 0.18) (Figure 7). High heterogeneity was observed (I² = 89%). Sensitivity analysis showed consistently high heterogeneity after exclusion of each of the individual studies.

Postoperative Complications

**Figure 8:**
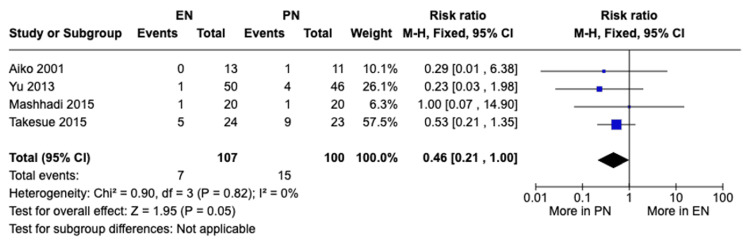
A forest plot comparing the incidence of pulmonary complications between EN and PN Hamai 2021 [15], Takesue 2015 [16], Mashhadi 2015 [17], Yu 2013 [18], Seike 2011 [19], Aiko 2001 [20] EN: Enteral nutrition; PN: Parenteral nutrition

Pulmonary complications: A meta-analysis of four studies with a total of 207 patients was done using a fixed-effects model. The total number of pulmonary complications was 22 (7 with EN and 15 with PN). There was a significant difference in pulmonary complications between both groups, with complications occurring more with patients receiving PN (RR 0.46, 95% CI [0.21, 1.00], P = 0.05) (Figure 8). No heterogeneity was present (I²=0%).

**Figure 9:**
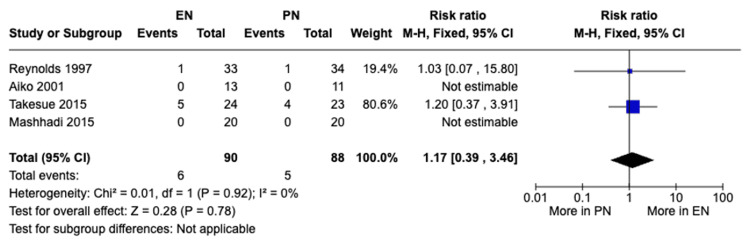
A forest plot comparing the incidence of anastomotic leakage between EN and PN Hamai 2021 [15], Takesue 2015 [16], Mashhadi 2015 [17], Yu 2013 [18], Seike 2011 [19], Aiko 2001 [20] EN: Enteral nutrition; PN: Parenteral nutrition

Anastomotic leakage: A meta-analysis of four studies with a total of 178 patients showed no statistically significant difference in anastomotic leakage rates in the EN and PN group using a fixed-effects model; five cases were reported in patients receiving EN while six cases in patients receiving PN (RR 1.17, 95% CI [0.39, 3.46], P = 0.78) (Figure 9). No heterogeneity was observed (I²=0%).

**Figure 10:**
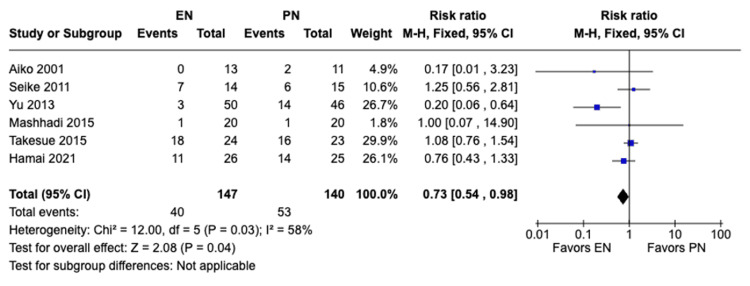
A forest plot comparing total postoperative complications between EN and PN using the fixed-effects model Hamai 2021 [15], Takesue 2015 [16], Mashhadi 2015 [17], Yu 2013 [18], Seike 2011 [19], Aiko 2001 [20] EN: Enteral nutrition; PN: Parenteral nutrition

**Figure 11:**
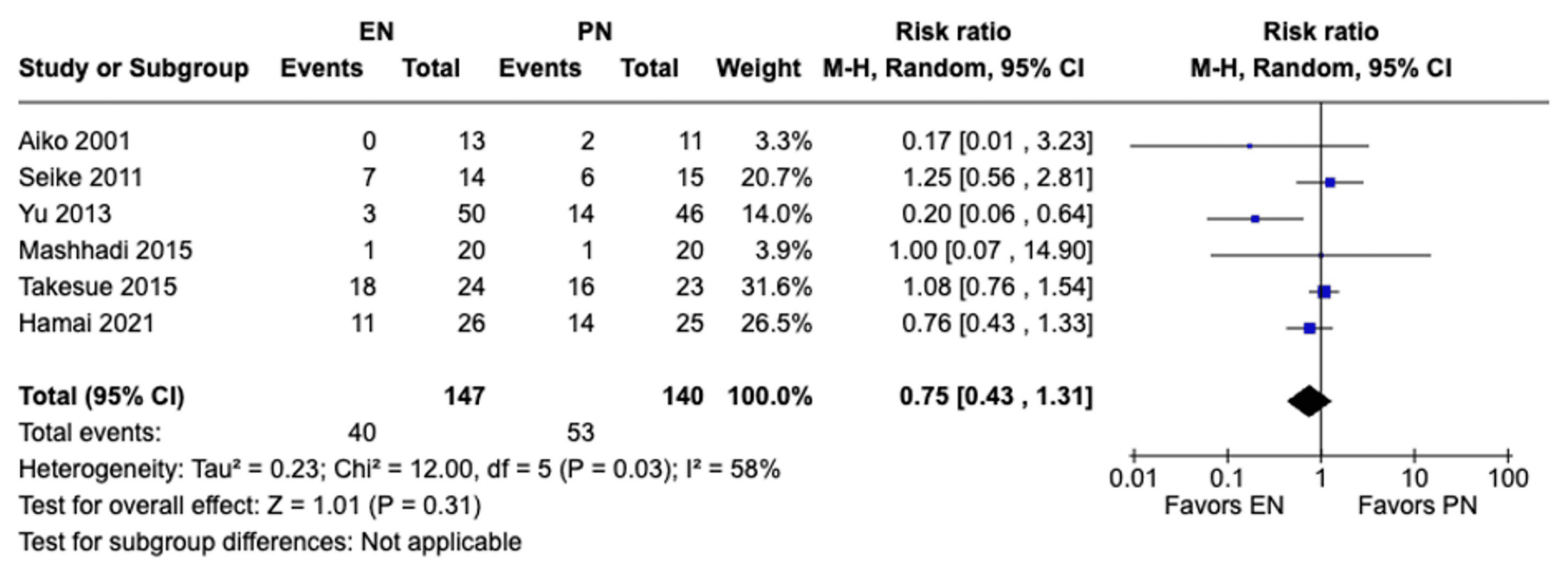
A forest plot comparing total postoperative complications between EN and PN using the random-effects model Hamai 2021 [15], Takesue 2015 [16], Mashhadi 2015 [17], Yu 2013 [18], Seike 2011 [19], Aiko 2001 [20] EN: Enteral nutrition; PN: Parenteral nutrition

Total postoperative complications: A meta-analysis of six studies with a total of 287 patients was done using both random and fixed-effects models. A total of 93 complications (40 in EN and 53 in PN) were observed. The fixed model showed a statistically significant difference in total postoperative complications with complications occurring more in patients receiving PN compared to those receiving EN (RR 0.73, 95% CI [0.54, 0.98], P = 0.04) (Figure 10). On the other hand, the random-effects model showed no significant difference in total postoperative complications between the two methods of feeding (RR 0.75, 95% CI [0.43, 1.31], P = 0.31) (Figure 11). Moderate heterogeneity was observed (I² = 58%). Sensitivity analysis showed that the study by Yu et al. [18] (2013) was the source of heterogeneity, which upon removal, resulted in no heterogeneity in our meta-analysis (I²=0%). Redoing the meta-analysis after exclusion of this study resulted in statistically non-significant differences.

Hospital Outcomes

**Figure 12:**
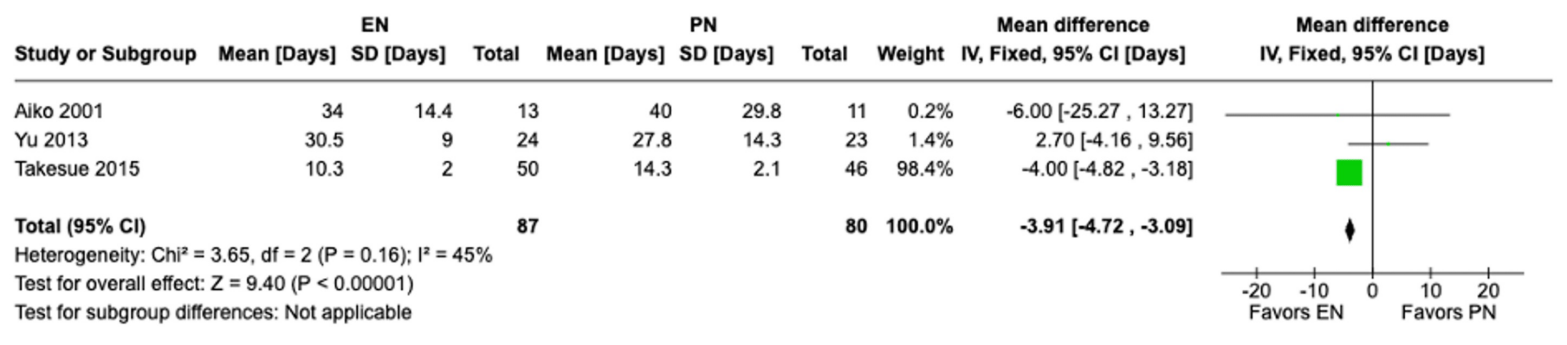
A forest plot comparing the postoperative hospital stay duration between EN and PN Hamai 2021 [15], Takesue 2015 [16], Mashhadi 2015 [17], Yu 2013 [18], Seike 2011 [19], Aiko 2001 [20] EN: Enteral nutrition; PN: Parenteral nutrition

Postoperative hospital stay: A meta-analysis of three studies with a total of 167 patients was done using the fixed-effects model. Results have shown a significant difference in postoperative hospital stay, with patients receiving EN having statistically significant shorter hospital stay durations compared to those receiving PN (MD -3.91, 95% CI [-4.72,-3.09], P<0.00001) (Figure 12). Heterogeneity tests showed low heterogeneity (I²=45%).

**Figure 13:**
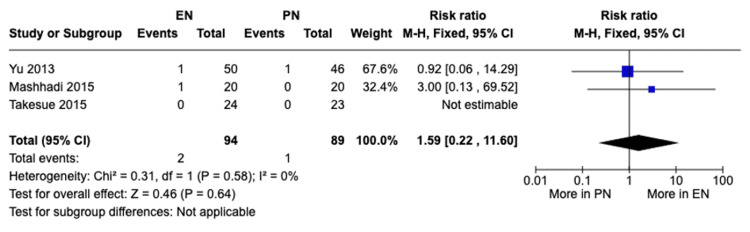
A forest plot comparing mortality rates between EN and PN Hamai 2021 [15], Takesue 2015 [16], Mashhadi 2015 [17], Yu 2013 [18], Seike 2011 [19], Aiko 2001 [20] EN: Enteral nutrition; PN: Parenteral nutrition

Mortality: A meta-analysis of three studies with a total of 183 patients showed no statistically significant difference in mortality between the EN and PN groups (RR 1.59, 95% CI [0.22, 11.60], P = 0.64) (Figure 13); two mortalities were reported in patients receiving EN while 1 was reported in patients receiving PN. No heterogeneity was observed (I²=0%).

Discussion

Overview of Findings

This meta-analysis aimed to evaluate the impact of EN and PN on post-operative outcomes in esophageal cancer patients post-esophagectomy. We included six studies with a total of 276 patients with variable sample sizes. The outcomes assessed in our study included albumin levels, prealbumin levels, total protein levels, bilirubin levels, C-reactive protein levels, pulmonary complications, anastomotic leakage, total postoperative complications, postoperative hospital stay, and mortality. Our findings demonstrated that EN is associated with a significant decrease in albumin levels. However, no significant differences were found between EN and PN with regard to prealbumin or total protein levels. Our analysis also revealed that PN was linked to higher postoperative levels of bilirubin. Additionally, EN showed benefits in reducing hospital stays, pulmonary and total postoperative complications; however, no significant differences were found in CRP levels, anastomotic leakage, or mortality rates between EN and PN.

Study Limitations

Some limitations were present in this meta-analysis and should be acknowledged. First, the sample sizes of the included studies were relatively small, ranging from 24 to 96 participants. While the overall pooled analysis was able to detect statistically significant differences in some outcomes, the limited sample sizes in some studies may have reduced the statistical power in other outcomes as well as generalizability of the results. Second, one study was assessed to have a high risk of bias due to missing outcome data, as multiple patients with complications were excluded, which may limit the applicability of the findings to all patients undergoing esophagectomy. This exclusion may have introduced bias, particularly in relation to postoperative outcomes, as patients with complications represent a clinically relevant group. Third, high heterogeneity (I² > 75%) was observed in some of the variables analyzed. Although sensitivity analyses were conducted, the heterogeneity remained high in those variables and no specific factors could be identified to explain the difference. The persistent heterogeneity may reflect differences in patient populations, interventions, or outcome definitions across studies, which could limit the comparability of the results. Subgroup and meta-regression analyses could not be performed given the limited sample sizes. Fourth, in cases where the standard deviation of changes from baseline was not reported, we imputed these values using a correlation coefficient of 0.99. While this approach was justified based on the controlled study conditions and a short follow-up period, it introduces an assumption that may not be entirely applicable across all studies. However, sensitivity analyses with multiple correlation coefficients confirmed the robustness of our findings under various assumptions.

Finally, the included studies were conducted in different countries at different times, which may introduce variability due to differences in healthcare standards, postoperative care, and clinical decision-making processes, such as discharge or any other criteria. These differences could affect the generalizability of the results to broader populations. We did not perform an assessment for publication bias, as the small number of studies (n = 6) would make such an analysis less meaningful. Nonetheless, the possibility of publication bias cannot be entirely excluded.

Nutritional Markers

In our meta-analysis, we found that patients receiving EN experienced a statistically significant greater decrease in albumin levels at POD 7 from baseline compared to PN (MD 0.12, 95% CI [0.00, 0.25], P = 0.05). This suggests that enteral nutrition might be associated with a greater decrease in albumin levels postoperatively, which is a marker of nutritional status and recovery [21]. This could indicate that PN provides more adequate nutrition than EN post-operatively; however, more investigations are needed to confirm its clinical significance. Conversely, there was no significant difference in changes in prealbumin levels (MD 0, 95% CI [-0.27, 0.27], P = 0.99) or total protein levels (MD 0.10, 95% CI [-0.04, 0.24], P = 0.15) from preoperative baseline to POD 7 between the EN and PN groups. This can mean that EN and PN may provide similar nutrition and may have similar effects on these markers in the early post-operative period. Additionally, it can also indicate that these markers might be less sensitive in detecting nutritional differences between EN and PN. These findings imply that EN and PN might have similar nutritional values and emphasizes the need for clinicians to consider the specific needs of their patients when choosing between EN and PN for post-operative nutrition.

Bilirubin and CRP Levels

In our meta-analysis, we also found that patients receiving PN experienced a greater increase in bilirubin levels when compared to the EN group at POD7 from baseline (MD -0.69, 95% CI [-1.12, -0.26], P = 0.002). These findings suggest that patients receiving PN may have greater increases in bilirubin post-operatively. Elevated levels of bilirubin can indicate liver disease such as hepatitis, cirrhosis, or biliary obstruction, as bilirubin is processed by the liver where it is conjugated and excreted into bile [22]. EN stimulates gut function and promotes bile flow; on the other hand, PN bypasses the gastrointestinal system and can lead to reduced bile flow. This reduction in bile flow impairs bilirubin metabolism and can lead to higher levels of post-operative bilirubin [23]. Our findings support these data and we can possibly conclude that parenteral nutrition will lead to higher post-operative bilirubin levels due to its effect on bilirubin metabolism and gut health. Clinicians should consider liver function tests as a factor when choosing between different postoperative nutrition strategies such as EN or PN, and our study recommends the use of EN to promote bile flow and improved liver function. Additionally, patients receiving PN were shown to have a higher increase in C-reactive protein levels compared to patients receiving EN, although not statistically significant (MD -1.35, 95% CI [-3.34, 0.64], P = 0.18). Previous studies have shown that EN preserves gut health and function; on the other hand, PN may cause mucosal gut atrophy and bacterial translocation leading to an increase in inflammatory cytokines and acute phase proteins such as CRP [23]. The randomized controlled trial conducted by Mashhadi et al. found that CRP was significantly lower in patients receiving EN compared to PN postoperatively [17]. Our findings suggest that EN might be a better option for less inflammation postoperatively.

Complications and Postoperative Hospital Stay Duration

Previous studies have shown that EN can preserve gut health, decrease bacterial translocation, and modulate the body’s immune response. Those effects of EN can lead to less complications and shorter hospital stays for patients postoperatively [18,23]. Similar studies such as the meta-analysis conducted by Peng et al. [24] and the meta-analysis conducted by Elke et al. [25] found that EN was beneficial in reducing complications and shortening postoperative hospital stay; however, there was no significant difference in mortality rate between EN and PN. In our meta-analysis, there was a significant difference in total postoperative complications (RR 0.73, 95% CI [0.54, 0.98], P = 0.04) and pulmonary complications (RR 0.46, 95% CI [0.21, 1.00], P = 0.05) between both groups, with complications occurring more with patients receiving PN. Also, there was a significant difference in postoperative hospital stay, with patients receiving EN having significant shorter hospital stay durations compared to those receiving PN (MD -3.91, 95% CI [-4.72,-3.09], P<0.00001). Additionally, there was no significant difference between both groups with regard to anastomotic leakage (RR 1.17, 95% CI [0.39, 3.46], P = 0.78) and with regard to mortality (RR 1.59, 95% CI [0.22, 11.60], P = 0.64). Our findings were consistent with previous studies with regard to the benefits of EN in reduced complications and shorter hospital stays, as well as no significant difference in reduction of mortality rates. The previous benefits in EN can be attributed to its preservation of gut health and modulation of the body’s immunity as mentioned previously. We also believe that mortality rates between EN and PN may not show significant differences due to multiple factors such as the benefits mentioned might not be significant enough to alter mortality rates and proper management of complications in both groups can lead to similar mortality rates. However, further studies are recommended to explore the similar mortality rates in both groups. Although previous studies suggest that EN can lower anastomotic leakage postoperatively, our analysis has shown no significant difference between the EN and PN groups in the incidence of anastomotic leakage. This possible discrepancy can be attributed to the limitations mentioned in our study such as the small sample sizes of the individual studies and it can also be attributed to the variability of countries of the included studies which can cause variability in the outcomes due to differences in healthcare standards and other criteria. Overall, our findings suggest that EN may be more favorable to PN in decreasing postoperative complications and shortening the hospital stay duration.

## Conclusions

In conclusion, our findings indicate that EN is associated with a significant decrease in albumin levels, suggesting that PN may provide more adequate nutritional support. However, no significant differences were found between EN and PN with regard to prealbumin or total protein levels, implying that both groups may have similar effects on prealbumin and total protein levels. Our analysis also revealed that PN was linked to higher postoperative levels of bilirubin and CRP, which supports the notion that EN might be preferable for preserving hepatic function and immunity. Additionally, while EN showed benefits in reducing hospital stays, total complications, and pulmonary complications, no significant differences were found in anastomotic leakage or mortality rates between EN and PN. Given the limitations in our meta-analysis such as the small sample sizes, potential bias in a study, and high heterogeneity in some variables, further research is recommended to explore the reasons behind some outcomes such as the lack of significant differences in pre-albumin levels, total protein levels, anastomotic leakage, and mortality. Our current findings favor the early use of EN in esophageal cancer patients after esophagectomy.
